# The non-pathogenic Australian rabbit calicivirus RCV-A1 provides temporal and partial cross protection to lethal Rabbit Haemorrhagic Disease Virus infection which is not dependent on antibody titres

**DOI:** 10.1186/1297-9716-44-51

**Published:** 2013-07-08

**Authors:** Tanja Strive, Peter Elsworth, June Liu, John D Wright, John Kovaliski, Lorenzo Capucci

**Affiliations:** 1 Division of Ecosystem Sciences, Commonwealth Scientific and Industrial Research Organisation, Canberra ACT 2601, Australia; 2 Invasive Animals Cooperative Research Centre, University of Canberra, Canberra ACT 2601, Australia; 3 Department of Agriculture, Fisheries and Forestry, Robert Wicks Pest Animals Research Centre, Biosecurity Queensland, Toowoomba 4350, QLD, Australia; 4 Natural Resources Management Biosecurity Unit, Biosecurity South Australia, Adelaide, SA, Australia; 5 Institutto Zooprofilattico Sperimentale della Lombardia e dell’ Emilia Romagna, 25124, Brescia, Italy

## Abstract

The endemic non-pathogenic Australian rabbit calicivirus RCV-A1 is known to provide some cross protection to lethal infection with the closely related Rabbit Haemorrhagic Disease Virus (RHDV). Despite its obvious negative impacts on viral biocontrol of introduced European rabbits in Australia, little is known about the extent and mechanisms of this cross protection. In this study 46 rabbits from a colony naturally infected with RCV-A1 were exposed to RHDV. Survival rates and survival times did not correlate with titres of serum antibodies specific to RCV-A1 or cross reacting to RHDV, but were instead influenced by the time between infection with the two viruses, demonstrating for the first time that the cross protection to lethal RHDV infection is transient. These findings are an important step towards a better understanding of the complex interactions of co-occurring pathogenic and non-pathogenic lagoviruses.

## Introduction

The prototype of the genus Lagovirus within the family *Caliciviridae* is Rabbit Haemorrhagic Disease Virus (RHDV) [1]. RHDV causes mortality rates of up to 90% in European rabbits (*Oryctolagus cuniculus*), the only species susceptible to the virus. It causes necrotizing hepatitis of the liver, severe disseminated intravascular coagulation and multiple organ failure, and usually kills rabbits within 72 h [2-4]. RHDV was first reported in an Angora rabbit colony in China [5], although recent phylogenetic analysis suggests that pathogenic RHDV may have evolved several decades earlier, also in Asia [6,7]. In the past 25 years RHDV has spread amongst domestic and wild rabbits across the world, causing economic losses to the meat industry [8] and ecological damage in countries where wild rabbits are a vital part of the ecosystem [9].

Australia heavily relies on RHDV to control overabundant European rabbits that were introduced to the continent approximately 150 years ago and multiplied to plague proportions, causing severe damage to native vegetation and impacting on the meat and wool industry [10-12]. In 1996, RHDV was officially approved for release as a rabbit biocontrol agent in Australia [10], and was very successful in reducing rabbit numbers initially [13]. However, it did not kill rabbits very effectively in some areas of Australia, and in rabbits from these regions antibodies cross reacting to RHDV were found [14]. This lead to the hypothesis that related endemic caliciviruses were circulating in these rabbits, providing some level of cross protection to lethal RDHV infection [15,16]. Such a virus was recently identified in wild Australian rabbits [17] and was designated Rabbit Calicivirus Australia 1 (RCV-A1). Evolutionary analysis suggests that this virus arrived in Australia together with the first wild rabbits, approximately 150 years ago [18]. Pilot infection studies showed that RCV-A1 causes a non-pathogenic infection of the small intestine and is capable of providing partial cross protection to lethal RHDV infection [19], confirming RCV-A1 is hindering effective RHDV-mediated rabbit biocontrol.

RCV-A1 adds to the growing number of non-pathogenic lagoviruses related to RHDV that are phylogenetically distinct from RHDV [20] and that have been reported from Italy [21], France [22-24], England [25,26] and moderately pathogenic viruses from the United States [27] and Europe [28]. Notably, studies that experimentally tested the immunological cross protection to RHDV conveyed by the non-pathogenic caliciviruses revealed disparate and partly unexpected results. A pilot infection study conducted with RCV-A1 resulted in 50% surviving the RHDV challenge, although only very low numbers were used in this study (*n* = 4) [19]. In several earlier studies wild-caught Australian rabbits presumed to have antibodies to a then unidentified RCV-A1 were challenged with RHDV and the observed protection rates varied between 36% [15], 33% [29] and 52% [14]. In contrast, the first non-pathogenic lagovirus that was described by Capucci et al. in Italy proved to be 100% protective to lethal RDHV infection [21]. Surprisingly, Le Gall-Reculé et al. did not find any cross protection provided by a recently isolated non-pathogenic French lagovirus [23], although this virus is genetically much more similar to RHDV than the Australian RCV-A1. These divergent findings underline the need to better understand both the extent and the mechanisms of cross protection provided by non-pathogenic relatives of RHDV.

In addition, immunological cross protection contributes to the complex interplay between host and pathogen, and the processes of host-pathogen co-evolution. Australian rabbits are beginning to develop genetic resistance to RHDV [30], by mechanisms that are not completely understood. It has been shown that Histo-Blood Group Antigens (HBGAs) on the epithelial linings of the rabbit gastro intestinal tract act as attachment factors for RHDV [31]. HBGA’s are synthesised by the enzyme #1,2 fucosyltransferase, which in rabbits is encoded by three functional genes, Fut1, Fut2, and Sec1 that have undergone multiple events of gene conversion during evolution [32]. Recent work suggests that different RHDV strains bind preferentially to different HBGA ligands, and rabbits expressing weaker binding HBGA phenotypes are found with increased frequency in wild rabbit populations following RHDV outbreaks [33,34]. Therefore, if a wild rabbit population is partially protected from lethal RHDV infection by immunological cross protection from a non-pathogenic calicivirus, this may reduce the selective pressure towards genetically resistant rabbits with weaker binding HBGA phenotypes as a means to avoid lethal RHDV infection [33].

Shedding more light on the extent and mechanisms of the immunological cross protection conveyed by the different non-pathogenic rabbit caliciviruses that are co-circulating with RHDV in wild rabbit populations is therefore vitally important to fully understand the implications for rabbit control in Australia. Conversely, such knowledge could contribute to the selection of non-pathogenic virus strains that could help protect endangered wild rabbit populations from RHDV outbreaks in Europe. While commercially available vaccine have proven effective in commercial rabbitries, vaccination campaigns in wild rabbits are economically and logistically impracticable [20].

The aim of this study was to gain better understanding of the extent of the cross protection that previous RCV-A1 infection can confer to lethal RHDV challenge by exposing a large number (*n* = 46) of rabbits with RCV-A1 antibodies to RHDV. We further investigated if the cross protection is dependent on the titres of antibodies raised against RCV-A1 and cross reacting to RHDV. We furthermore assessed if the protection rates vary when animals are experimentally inoculated with RHDV or acquire the infection via contact transmission.

## Materials and methods

### Animals and experimental design

Fifty, twelve-week-old domestic rabbits were acquired from a commercial rabbit breeding facility where RCV-A1 was circulating and individually marked with ear tags. Blood samples were taken at arrival (week 0), and then at week 3, 4 and 8 and analysed for antibodies against RHDV. One rabbit died shortly after arrival due to unknown causes, but unrelated to infection with either RCV-A1 or RHDV. Three of the rabbits that were either seronegative or IgM positive to antibodies cross reacting to RHDV at arrival were euthanazed two weeks after arrival. Their duodenum, liver and bile were analysed for the presence of lagoviruses using a universal lagovirus PCR that detects both RCV-A1 and RHDV, as described previously [17]. Male (*n* = 15) and female rabbits (*n* = 31) were housed separately in two group pens. Both pens had a floor area of approximately 15 m^2^. Eight weeks post acquisition 26 rabbits (9 males and 17 females) were infected orally with 500 LD_50_ of a commercially available RHDV-preparation (Elizabeth McArthur Agricultural Institute, Menangle, Australia) and returned to the group-pens.

The aim of this study was to assess if cross protection is dependent on the titres of RCV-A1-induced antibodies cross reacting to RHDV, and if there is a difference depending on the infectious dose of RHDV received. Therefore, the rabbits were divided into two groups, one group was experimentally inoculated with a moderate dose of RHDV (500 LD_50_), and the second was placed into the same pens to acquire the infection via contact (Table 1), such that both the experimentally infected rabbits and contact rabbits had high, medium or no IgG antibody titres cross reacting to RHDV at week 0 (Additional file 1). For the first 24 h, infected and non-infected animals were kept separate by a divider in the group pen to avoid low-dose infection of the contact animals through inoculum-contamination on feeders and water bottles. On the second day the dividers were removed and infected and contact animals were allowed to mix again to allow true contact infection. Throughout the trial, rabbits had *ad libitum* access to oaten hay and commercial rabbit pellets, as well as water bottles and dishes.

**Table 1:** Experimental groups, mortality rates, survival times and fever responses of the 46 rabbits subjected to RHDV challenge following previous RCV-A1 exposure

**ID**	**Sex**	**Inoculation**	**Survival**	**Survival time [d]**	Adjusted survival time [d]*	Fever**	Duration of fever [h] ***
**Group 1**							
**< 8 weeks between RCV-A1 and RHDV infection**							
***18***	***m***	***infected***	***Y***			***Y***	
***11***	***f***	***contact***	***Y***			***N***	
***40***	***f***	***contact***	***Y***			***Y***	
7	m	infected	N	8.8		Y	41.0
29	m	infected	N	9.9		Y	5.0
31	f	infected	N	2.6		N	
37	m	infected	N	11.4		Y	15.0
36	m	contact	N	12.2	9.8	Y	13.0
**Group 2**							
**8-10 weeks between RCV-A1 and RHDV infection**							
***25***	***f***	***contact***	***Y***			***Y***	
20	F	infected	N	10.5		Y	14.0
38	m	infected	N	8.5		N	
50	f	infected	N	4.5		Y	14.5
28	m	contact	N	10.4	8.0	Y	15.0
39	m	contact	N	12.2	9.8	Y	4.5
44	f	contact	N	9.9	7.5	Y	15.0
47	f	contact	N	7.0	4.6	Y	22.0
**Group 3**							
**>10 weeks between RCV-A1 and RHDV infection**							
***32***	***f***	***infected***	***Y***			***Y***	
2	f	infected	N	4.5		Y	14.5
3	f	infected	N	3.4		Y	2.3
4	f	infected	N	2.6		Y	7.5
6	m	infected	N	7.4		Y	16.0
12	f	infected	N	4.4		Y	5.3
15	f	infected	N	4.6		Y	15.8
16	m	infected	N	7.3		Y	14.0
17	m	infected	N	8.3		Y	6.5
19	m	infected	N	3.2		Y	3.5
21	f	infected	N	2.5		Y	14.0
24	f	infected	N	2.2		N	
8	m	contact	N	7.0	4.6	Y	5.0
23	f	contact	N	6.1	3.7	Y	4.5
26	m	contact	N	6.2	3.8	Y	9.0
27	m	contact	N	9.3	6.9	Y	12.0
33	f	contact	N	11.0	8.6	N	
45	f	contact	N	4.2	1.8	Y	6.5
**Rabbits with unknown infection history**							
***30***	***f***	***infected***	***Y***			***N***	
10	f	infected	N	6.2		Y	6.5
14	f	infected	N	2.8		Y	11.5
35	f	infected	N	9.5		Y	32.0
48	f	infected	N	9.7		Y	11.0
49	f	infected	N	4.0		Y	3.0
1	f	contact	N	7.8	5.4	Y	12.0
5	f	contact	N	7.7	5.3	Y	9.5
34	f	contact	N	15.8	13.4	N	
42	f	contact	N	6.7	4.3	Y	9.5
43	f	contact	N	4.7	2.3	N	
46	f	contact	N	5.2	2.8	Y	5.0

Surviving animals are shown in bold italics.

*Adjusted survival time is −2.4 days for the contact infected animals.

** Temperature >40.5°C. *** Time between recorded onset of fever and death.

Following the RHDV challenge, rectal temperatures were monitored twice daily and body weights were recorded on a daily basis. In addition, rabbits were checked every four hours during the day to record the time of death as accurately as possible. When found dead, rectal temperatures were taken and used to extrapolate the time of death based on experimentally determined post mortem temperature decay profiles (P. Elsworth, unpublished data). Where temperatures had dropped to near room temperature (> 8 h post mortem), the time since death (t_d_) was calculated as time between when the rabbit was last seen alive and found dead (∆t) minus half the difference between ∆t and 8 h: t_d_ [h] = ∆t - ((∆t [h]-8)/ 2). A final blood sample was collected from each animal at point of death, and at days 13 and 30 post RHDV challenge from the surviving rabbits, to monitor seroconversion to RHDV. The trial was terminated 30 days after the RHDV challenge and all remaining rabbits were euthanazed.

In order to assess if the observed survival rates and survival times were influenced by the duration between the infection with the two viruses, rabbits were divided into groups according to the time of their seroconversion to RCV-A1 for additional analysis. All rabbits that were seronegative or equivocal for RCV-A1 antibodies at week 0, but seroconverted within the next three weeks (*n* = 8) were assigned to Group 1 (< 8 weeks between the RCV-A1 and RHDV infection). Group 2 (*n* = 8) contained animals that tested positive for RCV-A1 IgM antibodies at week 0 or 3 as well as one animal that tested negative for RCV-A1 IgG at week 0 but positive at week 3. The time between infections for Group 2 was estimated based on previous observations that IgM antibodies to RCV-A1 appear from day 3 post infection (pi) and are usually detectable for at least 2 weeks [19,35] (and unpublished data), the time of RCV-A1 infection of the IgM positive animals in group two was therefore inferred to have been approximately 8–10 weeks prior to RHDV challenge. This group also contained rabbits #20 and #50, who had anti-RCV-A1 IgM antibodies at week 3 although their serostatus at week 0 was uncertain (Additional file 1).

The third Group was formed by animals (*n* = 18) that were already seropositive when purchased and that had acquired the RCV-A1 infection at an unknown time before acquisition. The remaining animals (*n* = 12) that had lost their ear tags and showed no RCV-A1 IgM at any time were not included into analyses that required information about their time of seroconversion to RCV-A1 (Table 1), however they were included into analyses assessing possible correlations of serum antibody titres at the time of challenge with survival times and survival rates.

All procedures involving animals were carried out according to the “Australian Code of Practice for the Care and Use of Animals for Scientific Purposes” and were approved by the Australian Department of Agriculture, Fisheries and Forestry Community Access Animal Ethics Committee (#CA 2008/09/303).

### Detection of viral RNA

RNA was extracted using the RNEasy kit (Qiagen, Hilden, Germany) for tissue samples, and the Invitrogen PureLink viral RNA kit (Invitrogen, Melbourne, Australia) for serum and bile samples, as per the respective protocols provided by the suppliers. Established protocols were used for the universal lagovirus RT-PCR [17], and RHDV real time qRT-PCR [19]. RCV-A1 qRT-PCR was carried out as described previously [36], but with primers WAU-1 1 F (ACCCTACAACCAACACATCAGG) and WAU-1 1R (ATGCCTGAAGCCAAAATAAACA). These primers are highly specific to the RCV-A1 virus strain used in this study and do not bind RHDV.

### Serology

ELISAs were used for the detection of IgA, IgG and IgM antibodies to RHDV [21,37,38] and RCV-A1 [39] as previously described. Sera were tested in duplicates, starting at a 1:40 dilution and subsequent 4 fold dilution steps. Sera that were only qualitatively tested to be either seropositive or negative were only analysed at a 1:40 and 1:160 dilution, also in duplicates. As the isotype ELISAs for the two viruses are known to cross react to various degrees, a more specific competition ELISA (cELISA) for RHDV and a specific blocking ELISA for RCV-A1 [40] were also used, starting at a 1:10 dilution with subsequent 4 fold dilution steps.

## Results

Several attempts were made to purchase 50 seronegative, 12 week old un-vaccinated rabbits from vaccinated does. At the time the trial was conducted, no RCV-specific ELISAs was available, therefore assays originally developed for RHDV were used to assess the antibody status of the animals. The first batch was unsuitable as almost all rabbits had very high anti-RHDV antibody titres, indicating exposure to RHDV at a young age and subsequent seroconversion. The second batch had a high proportion (approx 60%) of rabbits with cross reactive antibodies to a putative benign virus, as inferred using the methods described by Cooke et al. [38]. However, as it was not known which RCV-A1 strain had caused seroconversion in these rabbits, this cohort of rabbits was equally unsuitable. The third group of 50 rabbits also had antibodies cross reacting to RHDV, however 17 animals were still seronegative, and one had IgM antibodies cross reacting to RHDV, indicating that a benign virus infection was currently circulating in the colony. This final group of rabbits was then used to study the effects on RHDV infection in a colony naturally infected with RCV-A1.

Three of the rabbits that were either seronegative or had IgM antibodies cross reacting to RHDV at arrival (#13, #22 and #41) were euthanazed at week 2 and their duodenum, liver and bile analysed for the presence of lagoviruses in an attempt to isolate the circulating RCV-A1 strain. One rabbit (#22) tested positive in the PCR, indicating an active infection with a previously unknown RCV-A1 strain. The new strain was designated RCV-A1 WAU-1. Its relationship to the originally published strain MIC 1–4 [17] has been described elsewhere [18]. One rabbit (#9) died shortly after arrival due to unknown causes, but unrelated to infection with either RCV-A1 or RHDV, as no viral RNA for either virus could be detected in the duodenum or liver, respectively.

At the time of arrival (week 0), 14 of the 46 experimental rabbits tested negative or equivocal in all three RHDV ELISAs (IgG, IgM and cELISA), and all except 5 developed antibodies cross reacting to RHDV within 8 weeks following arrival. As soon as specific ELISAs for RCV-A1 were available [39,40], the sera were re-analysed. Eight of the 46 animals were seronegative or equivocal for RCV-A1 IgG, IgA and IgM at week 0, and by week 4 all rabbits had seroconverted to RCV-A1 (Additional file 1). Unfortunately, 14 rabbits in the female group had lost their ear tags on the first day, after the initial blood sample was collected. Numbers were re-assigned to these animals according to their bodyweights, however as we could not be entirely certain that the numbers were re-assigned correctly, no serology data is available for week 0 for these animals.

Eight weeks post acquisition, 26 of the rabbits were perorally challenged with RHDV (Table 1) and placed back into the group pens, so that the remaining rabbits could acquire the infection via contact. The overall survival rate was 13% (6 of 46), and three of the survivors had received RHDV via direct inoculation and three via contact infection. Fever was detected in 83% of all rabbits (38/46), with 85% (34/40) of the non-survivors and 67% (4/6) of the surviving rabbits showing temperatures above 40.5°C. The time to onset of fever varied greatly between individuals, the average time between the first detection of fever and death of the animals however was consistently short (11.6 ± 8 h (Mean ± SD)) (Table 1).

Five of the six surviving rabbits seroconverted to RHDV at the end of the trial (Table 2), as indicated by high titres in the RHDV cELISA. One rabbit (#11) avoided infection with RHDV. The serum of this rabbit showed medium titres in the RHDV IgG ELISA, however in the absence of a positive RHDV c-ELISA, IgM and IgA result, the IgG titres are likely to be RCV-A1 antibodies cross reacting in the RHDV IgG ELISA. This rabbit also had no detectable fever response. Rabbit #30 also showed no fever, however this rabbit was just beginning to seroconvert to RHDV at day 30 post challenge (dpc), (Table 2). As temperatures were not monitored regularly beyond 13 dpc, a possible fever episode may therefore have not been detected in this rabbit. Of the six surviving rabbits two (#18 and #32) showed temporary weight loss (> 10% but < 20%) following their fever episode; these were the only two cases of weight loss > 10% of all 46 rabbits in this experiment. These two animals also had a clear, highly viscous mucous discharge from the rectum for one day following their fever episode.

**Table 2:** Serology (RHDV only) of rabbits surviving the RHDV challenge

				**Antibodies to RHDV**								**RHDV copies/μl bile**
				**IgM**		**IgA**		**IgG**		**cELISA**		
**ID**	**Sex**	**Inoculation**	Weight loss*	**13 dpc**	**30 dpc**	**13 dpc**	**30 dpc**	**13 dpc**	**30 dpc**	**13 dpc**	**30 dpc**	**30 dpc**
18	m	infected	Y	+	–	+	+	> 40960	> 40960	> 640	> 640	4.11E + 03
32	f	infected	Y	+	+	+	+	> 40960	> 40960	> 640	>640	1.55E + 04
40	f	contact	N	+	+	+	+	D	> 40960	320	> 640	5.23E + 04
25	f	contact	N	–	–	–	D	40	> 40960	20	> 640	2.07E + 01
30	f	infected	N	–	–	–	+	1280	5120	–	160	1.70E + 01
11	f	contact	N	–	–	–	–	–	1280	–	–	–

Titres are expressed as the reciprocal dilutions at which the sera tested positive.

* Weight loss >10%, dpc = days post RHDV challenge, – = negative, + = positive.

*D*, Doubtful.

Unexpectedly, no correlation was observed between survival rates or survival times and serum antibody titres at the time of RHDV challenge. Pearson’s Product Moment Correlation revealed low but insignificant levels of negative correlation (RHDV-IgG: t = −1.54, *p* = 0.1319; RCV-A1-IgG: t = −1.4, *p* = 0.1696; RCV-A1-IgA: t = −0.914, *p* = 0.3667 and RCV-A1-bELISA: t = −0.568, *p* = 0.5737). Similarly, there was no correlation between survival rates and any of the antibody titres (Chi-Squared test, data not shown).

Notably, the average survival times of all rabbits that died were unusually high, 7.1 days ± 3.3 (Mean ± SD). Survival times for contact-infected rabbits were longer (8.4 days ± 3.1 (Mean ± SD)) compared to experimentally inoculated rabbits (6.0 days ±3.1 (Mean ± SD)). This 2.4 day average difference reflects the delay in acquiring the infection from the infected rabbits that were shedding virus.

Both the infected as well as the contact animals died in two cohorts. The first cohort died between day 2 and 4 in the infected animals and day 4 to 7 in the contact animals. The second cohort died between day 7 to 11 in the infected, and day 9 to 12 in the contact animals, respectively. When the survival times for the contact animals were corrected by the average time delay it took them to acquire the infection (−2.4 days), a clear biphasic pattern was observed. Notably, the animals that died in the second cohort showed no signs of prolonged disease, but appeared healthy until they suddenly developed a fever and died.

**Figure 1:**
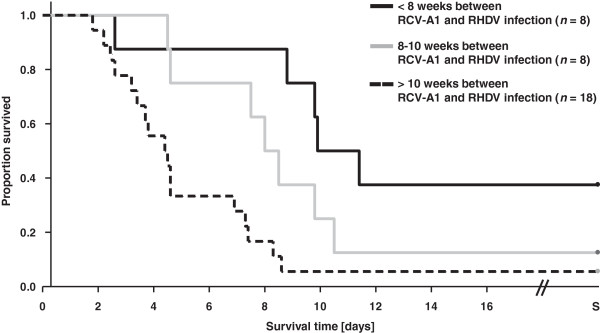
**Kaplan Meier survival analysis of rabbits challenged with RHDV.** Analysis was carried out using the SigmaPlot software version 12.3. S indicates survival until 30 days post RHDV challenge when the rabbits were euthanazed.

A high proportion of rabbits that had been seronegative to either RHDV or RCV-A1 when purchased and subsequently seroconverted within the following 8 weeks died in the second wave. This prompted us to investigate if the time between the infections with RCV-A1 and RHDV had an influence on survival rates and survival times, and animals were grouped according to the time between the two infections (Figure 1 and Table 1). In the group of the recently seroconverted animals (< 8 weeks between infections of RCV-A1 and RHDV) 3 of 8 rabbits survived (38%) and the animals in this group that did not survive showed prolonged survival times, with a median survival time of 9.9 days. The median survival time in the second group of animals with 8–10 weeks between infections with the two viruses was 8.0 days, and only one rabbit survived (13%). In comparison, median survival time in animals with a mature immune response in group three was 4.4 days, and in this group too only one animal survived (6%). In the Kaplan Meier survival analysis, the Logrank test was highly significant between group 1 and group 3 (*p* = 0.002) and significant between group 2 and group 3 (*p* = 0.038), but not significant between group 1 and group 2.

All rabbits that succumbed to RHDV infection had high levels of RHDV RNA in their bile (10^5^ to10^9^ copies per microliter) as determined by qRT-PCR, confirming RHDV as the likely cause of death (data not shown). Of the six surviving rabbits three had moderate viral loads of RHDV in the bile, while rabbits #25 and #30 had very low amounts of viral RNA just above the detection threshold, and rabbit #11 had no detectable RHDV RNA in the bile (Table 2). Trace amounts of RCV-A1 RNA just above the detection limit (1.4 × 10^1^ copies per microliter) were detected in the bile of rabbit #37 (data not shown), all other rabbits tested negative for RCV-A1 at time of death or euthanasia.

## Discussion

The most surprising finding of this study was that the partial protection provided by RCV-A1 to lethal RHDV infection does not depend on the titres of antibodies cross reacting to RHDV. This observation differs from results obtained in previous pilot infection studies, where the survival rate following RCV-A1 infection was 50%, and the surviving animals had higher antibody titres cross reacting to RHDV compared to the non-survivors [19]. However in that study only a small number of animals was used (*n* = 4). The data presented here show that the titres of serum antibodies both specific to RCV-A1 and cross reacting to RHDV are poor predictors for survival.

Instead, our results indicate that the timing of the RCV-A1 infection is likely to play a major role in the outcome of an RHDV challenge. There was a marked difference in the survival rates and survival times depending on the time between RCV-A1 and RHDV infection. As it was not known when the rabbits that were already seropositive at week 0 had acquired their RCV-A1 infection and serum samples were only collected at week 3, 4, and 8, it was only possible to assign the rabbits to three groups (Group 1: < 8 weeks; Group 2: 8–10 weeks and Group 3: > 10 weeks). Our results show that Group 1 rabbits with no more than 8 weeks between infections with the two viruses had the highest survival rates (38%) and longest survival times (median 9.9 days). Protection rates decreased to 13% in Group 2, while the survival times in this group were still unusually long with a median of 8 days. In contrast, for Group 3 rabbits with a mature immune response to RCV-A1 and at least 10 weeks between infections with the two viruses, there was no detectable protective effect against lethal RHDV infection. Survival rates of 6% and median survival times of 4.4 days are comparable to previous reports describing RHDV infections in naïve rabbits [2].

The temporal nature of the cross protection observed for RCV-A1 may help to explain the wide variations described for protection rates that the various non-pathogenic caliciviruses provide to RHDV challenge. Capucci et al. described complete protection (16/16 survived) when rabbits were challenged with RHDV, 24 days after infection with the Italian RCV [21]. Fifty percent protection (2/4 survived) was observed when rabbits were challenged with RHDV 24 days after infection with the Australian RCV-A1 [19] (and Table 3), and this reduced protection rate was initially ascribed to the lower degree of genetic relatedness and amino acid homologies to RHDV. Interestingly, Le-Gall and colleagues found no protection from RHDV challenge (1/15 survived) following previous infection with a recently discovered French non-pathogenic virus strain, although this European isolate is genetically more closely related to RHDV than RCV-A1 [23]. However, the rabbits subjected to RHDV challenge in their study were deliberately selected to exhibit high antibody titres cross reacting to RHDV, indicating mature antibody responses. Indeed, several of their rabbits had already been seropositive when acquired 3 weeks prior to challenge [23]. Our findings suggest that the window of heightened resistance to lethal RHDV infection may have been missed in their study. Future studies investigating the interference of pathogenic and non-pathogenic lagoviruses should take into account the time between infections as an important contributing factor to the varying degrees of cross protection.

**Table 3:** Summary of experiments assessing the level of cross protection to lethal RHDV infection provided by RCV-A1

**Study No.**	**Protection rate**	**N**	**RCV-A1 infection**	**Time post RCV-A1 infection**	**RHDV challenge**	**Reference**
1	52%	11/21	Natural infection	Unknown	One dose 1000 LD _50_, IM	[14]
2	36%	22/61	Natural infection	Unknown	One dose 1500 LD _50_, PO	[15]
3	33%	25/77	Natural infection	Unknown	One dose 1500 LD _500_, PO	[29]
4	50%	2/4	Experimental infection	4 weeks	One dose 500 LD _50_, PO	[19]
5a	38%	3/8	Natural infection	< 8 weeks	One dose 500 LD _50_, PO and/or continuous exposure (contaminated environment)	This report
5b	13%	1/8	Natural infection	8 - 10 weeks	One dose 500 LD _50_, PO and/or continuous exposure (contaminated environment)	This report
5c	6%	1/18	Natural infection	> 10 weeks	One dose 500 LD _50_, PO and/or continuous exposure (contaminated environment)	This report

*IM*, Intra muscular, *PO*, Peroral.

As the timing of previous RCV-A1 infection appears crucial for the outcome of subsequent RHDV challenge, an accurate estimate of overall protection rates conveyed by RCV-A1 is difficult to make. In addition, our data and previously published observations suggest that a variety of factors, such as the dose of RHDV, may play an additional role in the outcome of RHDV challenge. Table 3 provides a comparison of the current results with previously published data on experimental RHDV challenges of rabbits that were either known to be previously infected with RCV-A1, or inferred to be RCV-A1 positive due to antibodies cross reacting to RHDV. Protection rates of 33% to 52% were observed in animals experimentally infected with one dose of RHDV, ranging from 500 to 1500 LD50, applied via the PO or IM route (Table 3, study 1–4) [14,15,19,29]. In contrast, the current study (Table 3, study 5a, 5b and 5c) was conducted as a pen trial where rabbits were housed in large groups, and were both experimentally infected and acquired the infection via contact. It has been shown that high amounts of RHDV-RNA can be detected in rabbit faeces shortly before they die [19], and it is therefore likely that in this environment, over time, rabbits were exposed to continuously increasing quantities of virus due to virus shed by animals that succumbed to the challenge. In this trial, water dishes in particular appear to be a likely source of continuous exposure, as all rabbits used them and, although the water was changed daily, they were heavily soiled with faecal material.

It is unclear at this stage if the rabbits with the unusually long survival times avoided infection or disease. For example, rabbits #7, #17, #20, #37, #38 and #48 died between days 8 and 11 pi. As these animals were in the group of experimentally infected animals, the exact time between experimental infection and death was known. From day 3 post challenge onwards, these animals should have had a developing antibody response to RHDV. No increases in antibody titres reacting to RHDV were detected in these animals at the time of death (data not shown), however the viral load in the serum and bile was very high (10^9^ to 10^11^ and 10^5^ to 10^9^ RNA copies/mL, respectively) as determined by qRT-PCR (data not shown). It is possible that this high viremia neutralised moderate titres of early serum antibodies, resulting in negative ELISA results, although a successful adaptive response to RHDV usually results in effective clearance of infection. Alternatively, the initial experimental infection was unsuccessful and the rabbits acquired the virus later from the contaminated environment. Mucosal antibodies may have played a role in avoiding the infection but were not assessed in this study.

More research is needed to better understand the immunological mechanisms responsible for the temporal nature of the cross protection. Short-term elevation of non-specific innate immune mechanisms following RCV-A1 infection may lead to increased levels of infectious disease resistance in general [41]. Alternatively, more mature immune responses to RCV-A1 may be less protective to RHDV, due to affinity maturation. The selection of B-cells for production of antibodies with a higher affinity to the antigen (in this case RCV-A1) would lead to antibodies that are less cross reactive and thus possibly less protective to RHDV. However, the observation that antibody titres cross reacting to RHDV did not correlate with survival time or survival rates argues against this explanation.

Cellular immune mechanisms, which are known to be transiently elevated following infection, may also play an important role [41]. The S domain of the capsid protein VP60 that forms the inner shell of the viral particle [42] is highly conserved amongst lagomorph caliciviruses. RHDV and RCV-A1 have 93% amino acid identity in their inner shell domains, but only 73% amino acid identity in the antigenic P2 domains. It is likely that some antigens that are presented as MHC-I complexes are derived from the conserved S-domain of the capsid protein VP60 or from other non-structural proteins. The resulting cross reacting T-cell receptors may recognise RHDV infected cells and contribute to temporary suppression of productive RHDV infection, resulting in increased survival times. In some cases the infection may be slowed down sufficiently for the rabbit to develop a strong adaptive immune response specific to RHDV, leading to clearance of infection and ultimately survival. Similar mechanisms have been suggested for Influenza virus, where CD8+ T-cell responses following natural infection with Influenza A/H3N2 can induce heterosubtypic protection to avian influenza A/H5N1 [43].

The importance of T-cell responses in the protection from lethal RHD has also been discussed in the context of RHDV-vaccines, using RHDV specific antigens. Protection from VP60 expressed by a recombinant canarypox virus [44] or a recombinant ORF virus [45] to lethal RHDV was similarly independent from the vaccine induced antibody titres, and it was suggested that T-cell mediated immunity plays an important role [45]. However, commercial vaccines against RHDV that are based on inactivated, replication incompetent virus are effective in protecting rabbits against lethal RHD, and similarly recombinant VLPs also provide effective protection [46], although it has been demonstrated that VLPs alone are not effective in promoting the priming of naïve T-cells [47,48]. In addition, another study shows that adoptive transfer of polyclonal RHDV-positive serum can prevent lethal RHD in susceptible adult rabbits [49]. Future studies are clearly warranted shedding more light into the respective parts that cellular immunity, antibodies, but also immunogenetics [50] play in the protection and cross protection from lethal RHDV infection.

An additional intriguing result of this infection study was that it proved difficult to source seronegative rabbits that were devoid of antibodies cross reacting to RHDV due to a previous infection with benign calicivirus. Similar to murine norovirus, a calicivirus infecting mice that is non-pathogenic in the immunocompetent host and that was shown to be highly prevalent in scientific mouse breeding facilities tested [51], RCV-A1 may also be widespread amongst commercial rabbit breeding colonies. Non-pathogenic lagoviruses have been reported in European rabbitries [23], and more systematic testing of Australian rabbitries is clearly warranted to assess the prevalence of RCV-A1 in rabbits bred both for scientific purposes and for meat. Rabbit colonies may serve as a reservoir for RCV-A1, and transporting farmed rabbits or depositing soiled bedding material outside the breeding facilities is a potential mechanism facilitating the spread of RCV-A1.

The findings presented here have potentially important implications for rabbit biocontrol in Australia. The temporal nature of the cross protection conveyed by RCV-A1 indicates that there may be a window of opportunity for RHDV to work effectively as a biocontrol agent, provided that RCV-A1 is not present throughout the year. Studies are therefore needed to determine the infection dynamics and seasonal occurrence of RCV-A1 in Australia. In contrast, if non-pathogenic caliciviruses were to be considered as natural vaccines to RHDV in countries where rabbits are a valued wildlife species [9], the timing of the release of such strains would be crucial to ensure maximum benefits to biodiversity conservation.

## Competing interests

The authors declare they have no competing interests.

## Authors’ contributions

TS designed the study, wrote the manuscript and participated in conducting the animal experiments. PE participated in the design of the study, and conducted the animal experiments and statistical analysis. JL carried out and interpreted RCV-A1 immuno-assays. JDW carried out RCV-A1 immunoassays and participated in conducting the animal experiments. JK carried out RHDV immunoassays. LC participated in the study design, interpretation of results and provided key reagents. All authors have read and approved the final manuscript.

## Supplementary Material

Additional file 1 **RHDV and RCV-A1 serology of rabbits subjected to RHDV challenge following previous RCV-A1 exposure.** Minus (−) indicates a negative test result. D = equivocal. No animals had IgM antibodies at week 4 or 8. Animals surviving the RHDV challenge are shown in bold italics. Titres are expressed as the reciprocal dilutions at which the sera tested positive. Click here for file
