# Supplementary material for: The non-pathogenic Australian rabbit calicivirus RCV-A1 provides temporal and partial cross protection to lethal Rabbit Haemorrhagic Disease Virus infection which is not dependent on antibody titres
Source: Vet Res. 2013 Jul 8;44(1):51. doi: 10.1186/1297-9716-44-51 (PMC3733936; doi:10.1186/1297-9716-44-51)
Supplement: Additional file 1 — RHDV and RCV-A1 serology of rabbits subjected to RHDV challenge following previous RCV-A1 exposure. Minus (−) indicates a negative test result. D = equivocal. No animals had IgM antibodies at week 4 or 8. Animals surviving the RHDV challenge are shown in bold italics. Titres are expressed as the reciprocal dilutions at which the sera tested positive. [file 1297-9716-44-51-S1.pdf]

Rabbit ID	Antibodies cross reacting to RHDV							RCV-A1 antibodies													
	IgG				IgM		cELISA	IgG				IgM		IgA				bELISA			
	Week 0	Week 3	Week 4	Week 8	Week 0	Week 3	week 8	Week 0	Week 3	Week 4	Week 8	Week 0	Week 3	Week 0	Week 3	Week 4	Week 8	Week 0	Week 3	Week 4	Week 8
	**Rabbits culled for virus isolation**																				
13	-	-			-	D															
22	-	-			-	-															
41	-	80			pos	-															
	**Group 1**																				
	**< 8 weeks between RCV-A1 and RHDV infection**																				
11	***-***	***40***	***640***	***640***	***-***	***Pos***	***-***	***-***	***Pos***	***Pos***	***2560***	***-***	***Pos***	***-***	***2560***	***10240***	***2560***	***-***	***160***	***80***	***80***
18	***-***	***160***	***40***	***640***	***-***	***-***	***-***	***-***	***Pos***	***Pos***	***320***	***-***	***-***	***-***	***640***	***2560***	***-***	***-***	***20***	***40***	***10***
40	***-***	***-***	***80***	***40***	***-***	***-***	***-***	***D***	***Pos***	***Pos***	***320***	***-***	***-***	***-***	***10240***	***2560***	***-***	***10***	***20***	***20***	***10***
7	-	-	40	160	-	-	-	D	Pos	Pos	640	-	-	-	2560	2560	-	20	80	80	20
29	-	-	-	D	-	-	-	-	D	D	D	-	-	-	640	5120	-	-	20	20	-
31	-	D	D	-	-	-	-	-	Pos	Pos	640	-	-	-	2560	2560	-	-	40	20	-
36	-	D	80	640	-	-	-	-	Pos	Pos	2560	-	-	-	10240	5120	2560	D	80	80	80
37	-	-	40	160	-	-	-	-	Pos	Pos	2560	-	Pos	-	2560	10240	640	-	20	20	-
	**Group 2**																				
	**8-10 weeks between RCV-A1 and RHDV infection**																				
25	***160***	***640***	***40***	***40***	***-***	***-***	***-***	***Pos***	***Pos***	***Pos***	***320***	***Pos***	***-***	***2560***	***2560***	***640***	***-***	***80***	***80***	***80***	***20***
20	n.d.	-	-	160	n.d.	-	-	n.d.	pos	pos	1280	n.d.	Pos	n.d.	-	640	-	n.d.	80	80	40
28	-	D	D	40	-	-	-	D	Pos	Pos	80	Pos	-	1280	5120	2560	-	80	80	20	-
38	-	160	80	160	-	-	-	D	Pos	Pos	2560	Pos	-	40960	2560	5120	160	20	80	20	80
39	D	160	40	-	-	-	-	-	Pos	Pos	160	-	-	5120	10240	5120	640	20	20	20	10
44	-	-	40	320	-	D	-	D	Pos	Pos	640	Pos	Pos	2560	10240	5120	160	80	20	20	10
47	320	2560	1280	1280	-	-	-	Pos	Pos	Pos	2560	Pos	-	20480	10240	40960	2560	80	>160	>160	80
50	n.d.	-	-	160	n.d.	-	-	n.d.	-	D	640	n.d.	Pos	n.d.	640	2560	640	n.d.	20	20	20
	**Group 3**																				
	**>10 weeks between RCV-A1 and RHDV infection**																				
32	***2560***	***2560***	***5120***	***2560***	***-***	***-***	***10***	***Pos***	***Pos***	***Pos***	***2560***	***-***	***-***	***2560***	***2560***	***5120***	***2560***	***80***	***>160***	***>160***	***80***
2	20480	20480	10240	2560	-	D	-	Pos	Pos	Pos	5120	-	-	2560	2560	2560	1280	>160	>160	>160	80
3	640	5210	2560	2560	-	-	-	Pos	Pos	Pos	2560	-	-	640	2560	2560	1280	80	80	80	80
4	320	40	40	80	-	-	-	Pos	Pos	Pos	1280	-	-	640	640	160	-	-	-	-	-
6	2560	2560	2560	2560	-	-	-	Pos	Pos	Pos	2560	-	-	2560	2560	5120	2560	80	80	160	80
8	640	640	640	640	-	-	-	Pos	Pos	Pos	160	-	-	10240	10240	10240	1280	20	80	80	20
12	2560	1280	1280	10240	-	-	-	Pos	Pos	Pos	5120	-	-	10240	640	2560	2560	40	80	80	80
15	320	640	2560	640	-	-	-	Pos	Pos	Pos	320	-	-	10240	640	2560	-	80	20	20	-
16	-	-	-	-	-	-	-	Pos	Pos	Pos	640	-	-	-	640	2560	1280	-	20	40	10
17	160	640	320	640	-	-	-	Pos	Pos	Pos	160	-	-	2560	640	2560	1280	20	20	40	-
19	5120	5120	640	640	-	-	-	Pos	Pos	Pos	640	-	-	5120	640	640	-	160	80	80	20
21	160	1280	640	640	-	D	-	Pos	Pos	Pos	80	-	-	2560	40960	5120	5120	10	80	80	-
23	1280	5120	2560	2560	-	-	-	Pos	Pos	Pos	5120	-	-	5120	10240	640	2560	80	80	80	80
24	5120	5120	2560	2560	-	-	-	Pos	Pos	Pos	2560	-	-	2560	2560	640	-	80	80	80	20
26	-	D	40	160	-	-	-	Pos	Pos	Pos	320	-	-	2560	2560	640	-	20	20	20	-
27	160	D	D	80	-	-	-	Pos	Pos	Pos	160	-	-	2560	-	-	-	20	-	-	-
33	10240	10240	2560	2560	-	-	-	Pos	Pos	Pos	2560	-	-	2560	10240	10240	2560	80	>160	>160	80
45	10240	10240	10240	2560	-	-	-	Pos	Pos	Pos	5120	-	-	40960	10240	5120	2560	>160	80	>160	80
	**Rabbits with unknown infection history**																				
30	***n.d.***	***160***	***160***	***640***	***n.d.***	***-***	***-***	***n.d.***	***Pos***	***Pos***	***640***	***n.d.***	***-***	***n.d.***	***40960***	***10240***	***10240***	***n.d.***	***80***	***80***	***40***
1	n.d.	2560	2560	1280	n.d.	-	-	n.d.	Pos	Pos	2560	n.d.	-	n.d.	10240	640	-	n.d.	>160	20	20
5	n.d.	n	80	40	n.d.	-	-	n.d.	Pos	Pos	160	n.d.	-	n.d.	640	2560	-	n.d.	20	80	-
10	n.d.	D	40	40	n.d.	-	-	n.d.	Pos	Pos	640	n.d.	-	n.d.	640	2560	2560	n.d.	20	20	0
14	n.d.	40960	40960	40960	n.d.	-	-	n.d.	Pos	Pos	10240	n.d.	-	n.d.	10240	10240	10240	n.d.	>160	>160	80
34	n.d.	160	40	160	n.d.	-	-	n.d.	Pos	Pos	640	n.d.	-	n.d.	10240	2560	-	n.d.	160	80	20
35	n.d.	160	80	160	n.d.	-	-	n.d.	Pos	Pos	2560	n.d.	-	n.d.	160	320	-	n.d.	10	-	-
42	n.d.	640	320	320	n.d.	-	-	n.d.	Pos	Pos	640	n.d.	-	n.d.	10240	2560	640	n.d.	20	20	-
43	n.d.	-	-	-	n.d.	-	-	n.d.	Pos	Pos	320	n.d.	-	n.d.	640	640	160	n.d.	80	80	20
46	n.d.	640	640	640	n.d.	-	-	n.d.	Pos	Pos	2560	n.d.	-	n.d.	2560	640	-	n.d.	80	80	80
48	n.d.	-	-	640	n.d.	-	20	n.d.	Pos	Pos	1280	n.d.	-	n.d.	-	160	-	n.d.	20	40	20
49	n.d.	160	320	320	n.d.	-	-	n.d.	Pos	Pos	10240	n.d.	-	n.d.	2560	2560	640	n.d.	20	20	20

Minus (-) indicates a negative test result.
D= Equivocal; N.d. = not determined.
No animal had IgM antibodies at week 4 or 8.
Animals surviving the RHDV challenge are in bold italics.
